# Non-coding RNA in Fragile X Syndrome and Converging Mechanisms Shared by Related Disorders

**DOI:** 10.3389/fgene.2019.00139

**Published:** 2019-03-01

**Authors:** Yafang Zhou, Yacen Hu, Qiying Sun, Nina Xie

**Affiliations:** ^1^Department of Geriatric Neurology, Xiangya Hospital, Central South University, Changsha, China; ^2^National Clinical Research Center for Geriatric Disorders, Central South University, Changsha, China

**Keywords:** non-coding RNA, Fragile X syndrome, RNAi mediated epigenetic silencing, microRNA mediated translational regulation, potential converging mechanisms

## Abstract

Fragile X syndrome (FXS) is one of the most common forms of hereditary intellectual disability. It is also a well-known monogenic cause of autism spectrum disorders (ASD). Repetitive trinucleotide expansion of CGG repeats in the 5′-UTR of *FMR1* is the pathological mutation. Full mutation CGG repeats epigenetically silence *FMR1* and thus lead to the absence of its product, fragile mental retardation protein (FMRP), which is an indispensable translational regulator at synapsis. Loss of FMRP causes abnormal neural morphology, dysregulated protein translation, and distorted synaptic plasticity, giving rise to FXS phenotypes. Non-coding RNAs, including siRNA, miRNA, and lncRNA, are transcribed from DNA but not meant for protein translation. They are not junk sequence but play indispensable roles in diverse cellular processes. FXS is the first neurological disorder being linked to miRNA pathway dysfunction. Since then, insightful knowledge has been gained in this field. In this review, we mainly focus on how non-coding RNAs, especially the siRNAs, miRNAs, and lncRNAs, are involved in FXS pathogenesis. We would also like to discuss several potential mechanisms mediated by non-coding RNAs that may be shared by FXS and other related disorders.

## Introduction

Non-coding RNA is a kind of transcript from DNA but not meant for protein translation. Accumulating evidence has shown that non-coding RNA molecules play indispensable roles in diverse cellular processes. Understanding mechanisms mediated by these non-coding RNAs are of great importance for understanding the pathogenic process of relevant diseases. Functional molecules classified into this category have included micro-RNA (miRNA), long non-coding RNA (lncRNA), small-interfering RNA (siRNA), transfer RNA (tRNA), ribonucleoprotein RNA (rRNA), and piwi- interacting RNA (piwi-RNA), etc. (Cao et al., 2006; Beermann et al., 2016; Treiber et al., 2018).

Fragile X syndrome (FXS) is one of the most common forms of hereditary intellectual disability. It is also a well-known monogenic cause of autism spectrum disorders (ASD) (Wang et al., 2012). *FMR1* is the responsible gene. The repetitive trinucleotide expansion of CGG repeats in the 5′-untranslated region (5′-UTR) of *FMR1* is the pathological mutation. Normal individuals usually bear CGG repeat expansion ranging from 6 to 55, while in FXS patients, this expansion often reaches beyond 200, known as the full mutation (Santoro et al., 2012). The *FMR1* gene was first cloned in 1991, rendering FXS the first discovered disease caused by trinucleotide expansion mutation (Verkerk et al., 1991). In addition to this mutation, conventional mutations including gross deletions, small indels, and missense or nonsense mutations have also been reported (Luo et al., 2014, 2015; Myrick et al., 2014). Over the last decade, FXS caused by the full mutation CGG repeat expansion receives the most intense attention. The full mutation CGG repeats lead to epigenetic silencing of *FMR1* and absence of its product, fragile mental retardation protein (FMRP) (Xie et al., 2016). FMRP is a complex RNA binding protein which plays indispensable roles in synaptic plasticity. It has four important RNA binding motifs including one arginine-glycine-glycine (RGG box) and three K homology domains (KH0, KH1, and KH2), recognizing special RNA secondary structures, such as the kissing complex and G quadruplex (Darnell et al., 2001, 2005; Myrick et al., 2015). By binding with target mRNA, FMRP mainly functions as a translation repressor at synapsis, regulating local translation spatially and temporarily to shape synaptic structure and plasticity (Nakamoto et al., 2007; Bassell and Warren, 2008; Huang et al., 2014). Most of FXS patients’ phenotypes could be attributed to the loss of FMRP. Clinically, the most common symptom is intellectual retardation. Other neurological symptoms include ASD, attention deficit hyperactivity disorder (ADHD), and epilepsy. Non-neurological symptoms include macroorchidism, distinct facial features (elongated faces, protruded ears, and big forehead), and connective tissue abnormalities (mitral valve prolapse, flat fee, joint hyperextensibility, and high arched palate) (Jacquemont et al., 2007).

FXS is the first neurological disorder found to be linked to the miRNA pathway (Jin et al., 2004a). In this review, we mainly focus on how non-coding RNAs, especially the siRNAs, miRNAs, and lncRNAs, are involved in FXS pathogenesis. We would also like to discuss several potential mechanisms mediated by non-coding RNAs that may be shared by FXS and other related disorders.

## Non-Coding RNA Mediated Mechanisms in FXS Pathophysiology

### How RNAi Is Involved in the Epigenetic Silencing of *FMR1*?

RNA interference (RNAi) refers to the process of mRNA degradation or translation inhibition mediated by small RNAs. It is a mechanism regulating gene expression at the post-transcriptional level in eukaryotic cells. siRNA and miRNA are the two most important types of small RNAs involved. Although their origins are different, they share similar downstream machinery when encountered with Dicer, an RNase III-like enzyme initiating RNAi. Precursors of siRNA or miRNA are cleaved by Dicer to be short double-stranded RNAs (dsRNAs). In these duplexes, only one functional strand is kept, while the other one is degraded. The functional strand is the mature siRNA or miRNA that finally assemblies with Argonaute proteins (AGO) to form the RNA-induced silencing complex (RISC). A major difference lies in that siRNA requires perfect base-pairing with the target sequence to guide AGO to the targeted locus while miRNA could tolerate several mismatches. AGO associated with siRNA usually induces mRNA degradation, in contrast, AGO loaded with miRNA tends to cause translation inhibition (Ha and Kim, 2014; Holoch and Moazed, 2015; Hu et al., 2017).

Full mutation CGG expansion triggers extensive DNA methylation, repressive histone modification, and chromatin condensation in the 5′-UTR of *FMR1*, transcriptionally silencing the gene and leading to loss of FMRP (Coffee et al., 1999; Biacsi et al., 2008; Alisch et al., 2013). How are the abnormal epigenetic markers triggered and maintained? Although this epigenetic silencing process has been the focus of study over the last two decades, detailed mechanisms are still mysterious. The consensus is that full mutation CGG repeat is the prerequisite trigger to initiate and maintain the repressive epigenetic changes of *FMR1*. Several models have been indicated to date, albeit it is still difficult to form an integrated one. The first model is DNA based. Secondary structures formed by the CGG repeats serve as substrates for DNA methyltransferase to initiate *de novo* DNA methylation, or as targets bound by repeat binding proteins to recruit repressor complexes (Smith et al., 1994; Bulut-Karslioglu et al., 2012). The second model is RNA based, where hairpin structures in mRNA formed by CGG repeats exceeding a certain threshold trigger the RNAi pathway to deposit repressive epigenetic markers (Kim et al., 2006; Usdin et al., 2014). The third model is a blended one, where the DNA:RNA hybrid is at play. During transcription, hybridization of the nascent RNA to its unzipped DNA template forms a special R-loop, which may act as a structural block or nucleosome analogy to induce epigenetic silencing (Colak et al., 2014; Groh et al., 2014). Our discussion below is focused on the RNA based model. What may be the role of RNAi in the epigenetic silencing of *FMR1*?

RNAi has been suggested as a conservative mechanism participating in the formation of heterochromatin in fission yeast. In this scenario, the siRNA serves as a localizer for the RISC complex to achieve site-specific epigenetic modulation. RISC is associated with Ago1 (the yeast argonaute homolog), Chp1 (a protein required for methylation of H3K9), and Tas3 (a protein required for localization of the chromatin). This RISC-like heterochromatin-targeting complex is termed RITS (Reinhart and Bartel, 2002; Volpe et al., 2002; Verdel et al., 2004).

**Figure 1:**
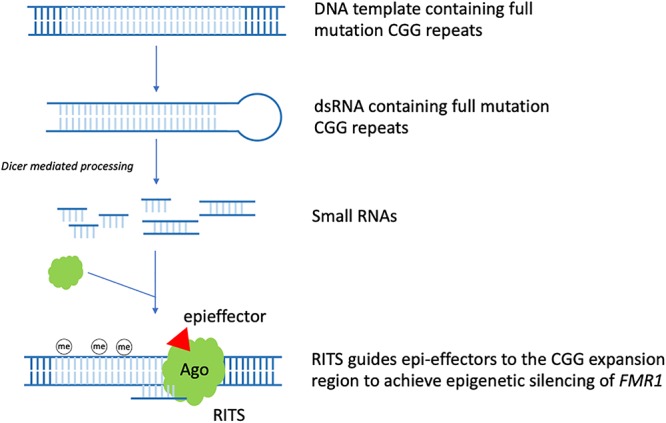
RNAi mediated *FMR1* epigenetic silencing. In early embryo development, *FMR1* gene containing the full mutation CGG repeats is being transcribed actively. Bi-directional transcription of the DNA template generates dsRNAs bearing full mutation CGG repeats. Dicer further cleaves these dsRNAs to produce small RNAs. After being incorporated into the RITS complex, similar to siRNA, these small RNAs guide RITS to the CGG expansion region, recruiting epi-effectors, such as histone methyltransferase and DNA methyltransferase, to induce epigenetic silencing of *FMR1* (the CGG expansion region is light blue colored).

In the context of FXS, it was observed that stable hairpin-structured RNAs containing pre-mutation CGG repeats could be processed by Dicer to generate small RNAs. Based on this observation and the role siRNA has in heterochromatin formation. A model for the RNAi mediated methylation of full mutation CGG repeats was proposed in 2004. In early embryo development, *FMR1* gene containing the full mutation CGG repeats is being transcribed actively. Bi-directional transcription of the DNA template generates dsRNAs bearing full mutation CGG repeats. Dicer further cleaves these dsRNAs to produce small RNAs. After being incorporated into the RITS complex, similar to siRNAs, these small RNAs guide RITS to the CGG expansion region, recruiting epi-effectors, such as histone methyltransferase and DNA methyltransferase, to induce *FMR1* silencing epigenetically (Jin et al., 2004a; Figure 1). Thus, RNAi may play a critical role in the epigenetic silencing process of *FMR1*.

### How miRNA Is Involved in FMRP Mediated Translational Regulation?

miRNA is a short non-coding single-stranded RNA molecule consisting of 20–25 nucleotides. Its biogenesis experiences a series of events. The Primary microRNA (pri-miRNA) was first converted to the precursor microRNA (pre-miRNA) by a microprocessor comprising of DGCR8 and DROSHA proteins. Next, Dicer cleaves the pre-miRNA to generate short dsRNAs, the functional strand of which further assemblies with AGO proteins (also known as PIWI/PAZ-domain proteins) to form the RISC complex. This functional strand is the mature miRNA. It functions as a localizer as well as a translation repressor by binding to the 3′-untranslated region (3′-UTR) of the mRNA targets (Carthew and Sontheimer, 2009; Holoch and Moazed, 2015).

FXS is the first neurological disease being linked to miRNA pathway dysfunction (Jin et al., 2004a). FMRP is a RNA binding protein which mainly functions as a translational repressor. Studies from several groups suggested that FMRP is associated with critical components of the miRNA pathway. Loss of FMRP may cause aberrant miRNA profiling (Caudy et al., 2002; Ishizuka et al., 2002). The hippocampus of post-natal day 7 *FMR1* knockout mice showed significantly different miRNA expression profiles compared to the WT (Liu et al., 2015). In drosophila, dfmr1 is associated with Ago1. Absence or partial loss of Ago1 impairs FMRP-mediated regulation of neural development and synaptogenesis (Jin et al., 2004b). Recently, in a FXS mice model, FMRP was shown to participate in pri-miRNA processing by upregulating DROSHA expression at the translational level. Loss of FMRP was associated with accumulation of specific pri-miRNAs and reduction of pre-miRNAs (Wan et al., 2017).

**Figure 2:**
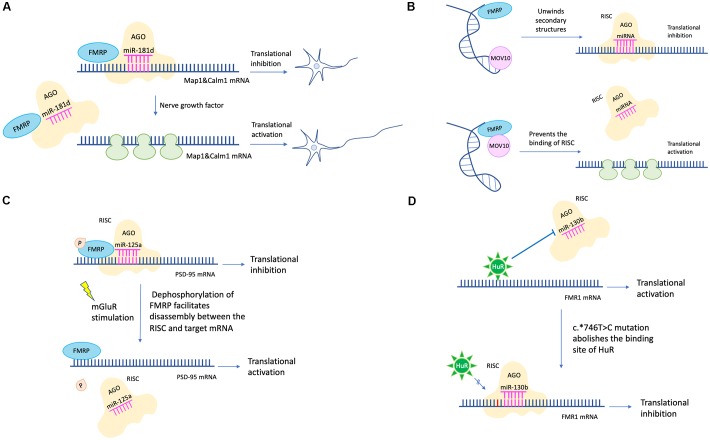
miRNA mediated translational regulation in FXS. **(A)** FMRP and miR-181d cooperatively regulate the axon elongation process by repressing translation of Map1and Calm1. Upon nerve growth factor stimulation, Map1 and Calm1 mRNAs are released from granules suppressed by FMRP and miR-181d to translate actively for axon elongation. **(B)** MOV10 has a dual function in translation regulation. For translational activation, MOV10 unwinds RNA secondary structures to expose the miRNA recognition element (MRE). The association between MRE and RISC is facilitated. For translational inhibition, FMRP and MOV10 bind to the target mRNA in proximity. The association between MRE and RISC is disrupted. **(C)** Phosphorylation of FMRP promotes assembly between miR-125a-AGO2 complex and PSD-95 mRNA. Upon mGluR stimulation, dephosphorylation of FMRP facilitates disassembly between miR-125a-AGO2 complex and PSD-95 mRNA. **(D)** The c.^∗^746T > C variant abolishes the binding between HuR and *FMR1* mRNA. Instead, miR-130b binds to the 3′-UTR of *FMR1* mRNA, resulting in decreased FMRP expression and FXS phenotypes (the c.^∗^746T > C variant was in red color).

Efforts have been made to clarify detailed mechanisms of how FMRP regulates protein translation via the miRNA pathway. For example, FMRP could stabilize the binding of miRNA to its target mRNA via the KH domain (Plante et al., 2006). Besides, partly dependent on miR-125b, FMRP negatively regulates the expression of NR2A, a NMDA receptor subunit affecting synaptic plasticity (Edbauer et al., 2010). Moreover, FMRP regulates axon guidance gene via the miRNA pathway by repressing the RE-1 silencing transcription factor (REST) (Halevy et al., 2015). FMRP and miR-181d cooperatively regulate the axon elongation process by repressing translation of Map1 (a microtubule-associated protein) and Calm1 (a calcium signaling regulator). Upon nerve growth factor stimulation, Map1 and Calm1 mRNAs are released from granules suppressed by FMRP and miR-181d to translate actively for axon elongation (Wang et al., 2015; Figure 2A).

Later studies in succession provided further insight into how FMRP represses target mRNA translation via RNAi. A possible mechanism is FMRP interacts with MOV10, a RNA helicase implicated in miRNA pathway. MOV10 has a dual function in translational control. It assists the miRNA-mediated translational repression for specific RNAs but inhibits others. In the former case, MOV10 binds to the 3′-UTR of target mRNA, unwinds specific RNA secondary structures, and exposes the miRNA recognition elements (MRE) to facilitate the interaction between MRE and RISC. In the latter scenario, both FMRP and MOV10 bind to the target mRNA in proximity. The association between MRE and RISC is disrupted (Kenny et al., 2014; Figure 2B). Another mechanism is that FMRP utilizes miRNA to control translation at synapses temporally and spatially. For example, phosphorylation of FMRP promotes assembly between miR-125a-AGO2 complex and PSD-95 mRNA. Upon mGluR stimulation, dephosphorylation of FMRP facilitates disassembly between miR-125a-AGO2 complex and PSD-95 mRNA (Muddashetty et al., 2011; Figure 2C).

Endogenous FMRP expression level is also regulated by the miRNA pathway. For example, the expression level of miR-130b was negatively correlated with the FMRP level in mice embryonic neural precursor cells (NPC) (Gong et al., 2013). In humans, miR-130b also seems to be a negative regulator of FMRP. Utilizing a patient-derived cell line, researchers identified a RNA binding protein HuR which may function as a RISC antagonist, as its binding site overlaps with that of miR-130b. This patient has no full mutation, however, the c.^∗^746T > C variant in 3′-UTR renders him with significantly decreased FMRP level and FXS phenotypes. A likely mechanism is that the c.^∗^746T > C variant abolishes the binding between HuR and *FMR1* mRNA. Instead, miR-130b binds to the 3′-UTR of *FMR1* mRNA, resulting in decreased FMRP expression (Suhl et al., 2015; Figure 2D). Additionally, in a zebrafish FXS model, researchers transgenically overexpressed rCGG (CGG trinucleotide repeats) motif via high titer retroviral delivery. As the expression level of rCGG increased, the level of *FMR1*-miRNA was increased, oppositely, the level of *FMR1* transcription was decreased, indicating that rCGG derived-miRNA participated in *FMR1* transcription suppression (Lin, 2015).

In sum, miRNA facilitates the FMRP mediated translational regulation via diverse mechanisms to shape synaptic plasticity and morphology, to achieve translational control temporally and spatially, and to regulate endogenous FMRP level (Figure 2).

### lncRNA Participates in FXS Pathophysiology

Long non-coding RNA (lncRNA) is a genome transcript longer than 200nt. As a major category of non-coding RNA, lncRNA participates in formation of RNA-protein complexes, gene regulation, modulation of protein localization, and X chromosome inactivation (Wu R. et al., 2016). In FXS, lncRNA may affect the cell proliferation process and may serve as novel clinical biomarkers. Similar to miRNA and siRNA, its role in FXS pathogenesis should not be ignored.

FMR4 is a 2.4 kb lncRNA, which is primate-specific and plays an anti-apoptotic role in human cells. It is transcribed from *FMR4*, which resides upstream *FMR1*. *FMR4* and *FMR1* a bi-directional promoter. In FXS, both of them are silenced due to the CGG repeat expansion, which may contribute to FXS pathogenesis (Khalil et al., 2008). FMR4 is also a trans-acting element which regulates hundreds of genes involved in neural development. By altering chromatin state epigenetically, FMR4 selectively modulates the cellular proliferation of human NPC (Peschansky et al., 2016). Later on, combining the technology of rapid amplification of cDNA ends (RACE) and next generation sequencing, researchers investigated the *FMR1* gene locus systemically and found two novel lncRNAs, FMR5, and FMR6. FMR5 is transcribed in the sense direction, the transcription start site (TSS) of which resides upstream the *FMR1* TSS. FMR6 is transcribed in the antisense direction, beginning from the 3′-UTR of *FMR1*. FMR5 and FMR6 may be novel clinical biomarkers due to their distinct expression patterns between full mutation and premutation patients (Pastori et al., 2014). A recent study provides new clues in how lncRNA is involved in FXS pathophysiology. TUG1 is a lncRNA which prevents axonal growth by negatively regulating the SnoN-Ccd1 pathway. In WT mice, FMRP directly binds with TUG1 to decrease its stability. However, in FXS mice, absence of FMRP leads to TUG1 overexpression. TUG1 interacts with SnoN, a crucial transcriptional regulator which controls axonal growth via regulating the actin-binding protein Ccd1. By affecting the transcriptional activity of SnoN, overexpression of TUG1 decreases the level of Ccd1 mRNA and protein, leading to abnormal axonal growth (Guo Y. et al., 2018). Albeit reference regarding this section is scarce, the importance of lncRNA is non-negligible, as evidenced by the research findings described above.

### miRNA Links FMRP to Glia Function

Glutamate is a major excitatory neurotransmitter in brains. Dysregulation of glutamate in neurons participates in various neuropsychiatric disorders by affecting synaptic plasticity. Interestingly, abnormal regulation of glutamate in cells surrounding neurons may also be an important pathogenic process. For the encircled neurons may become more exciting. For example, in the astrocytes isolated from the cortex of *FMR1*-deficient mice, the GLT1 expression, a major glutamate transporter of glia, is significantly reduced compared to the WT, so is the uptake of glutamate. Additionally, treating the cortical slices with GLT1 inhibitors results in significantly enhanced neuronal excitability in *FMR1*-deficient mice but not in controls. Moreover, based on an astroglia-specific conditional FMRP knockout and restoration mice model, researchers found that selective re-expression of FMRP in astrocytes of *FMR1*-deficient mice rescues phenotypes of the decreased GLT1 expression in cortical astrocytes and the abnormal spine morphology in cortical pyramidal neurons. Upregulation of GLT1 expression alleviates enhanced neuronal excitability and corrects spine abnormality in *FMR1*-deficient mice. These results together indicate that absence of FMRP may impair the ability of astrocytes, via dysregulated GLT1, to remove excessive glutamate from neurons and thus lead to neuronal hyperexcitability in FXS (Higashimori et al., 2013, 2016).

Notably, the regulation of GLT1 may be mediated by exosomes. Exosomes are membrane vesicles secreted by cells. They contain various signaling molecules including miRNAs. After being secreted into extracellular space, exosomes could be fused into surrounding cells to assist intercellular communication (Xiao et al., 2017). miR-124a has a positive effect on the expression level of GLT1 both *in vivo* and *in vitro*. Transfer of miR-124a from neurons to astrocytes via exosomes selectively increases the astroglial GLT1 protein level without affecting the mRNA level, suggesting the exosome-derived miR-124a is a regulator of the GLT1 expression (Morel et al., 2013).

It is likely that absence of FMRP may cause aberrant miRNA function in the neuron/astroglia interaction and thus affect GLT1 expression and glutamate uptake ability of astrocytes. More studies are needed to shed light on this topic.

## Non-Coding RNA Mediated Mechanisms Shared by FXS and Related Disorders

### miRNA of FXTAS May Also Be Pivotal to FXS

Fragile X tremor ataxia syndrome (FXTAS) is an aging-related neurodegeneration disorder, featured by progressive tremor, ataxia, and cognition decline. In FXTAS, *FMR1* containing the premutation CGG repeat expansion (60–200 repeats) is not silenced. Instead, it is transcribed actively, resulting in significantly higher mRNA level than normal but less FMRP protein. It is thought that the gain-of-function RNA toxicity is to blame, of which the miRNA pathway is at play. For instance, in neuronal cells and brain tissues from FXTAS patients, RNA aggregates containing pre-mutation CGG repeats decrease mature miRNA levels by sequestering RNA-binding proteins which are crucial to miRNA biogenesis, such as DGCR8 and DROSHA (Sellier et al., 2013). In olfactory neurons of *C. elegans*, expression of the expanded CGG repeats weakens the olfactory plasticity formation by interacting with the *C. elegans* specific argonaute ALG-2 (Juang et al., 2014). Additionally, miRNA-277, 424, 101, 129-5p, and 221 have also been indicated in the pathogenesis of FXTAS (Tan et al., 2012; Alvarez-Mora et al., 2013; Zongaro et al., 2013).

FXTAS is the neurodegenerative disorder most closely related to FXS. FXTAS and FXS may have overlapping mechanisms in the earlier neurodevelopmental stage or the later neurodegenerative stage, as either the premutation or the full mutation *FMR1* is transcribed actively at the very beginning, and both of them could lead to cognition impairment in late-life. miRNAs that are critical to FMRP function in FXTAS may also be pivotal to FXS. Discovery of such miRNAs is necessary.

### miRNA Regulating mTOR Activity Might Play a Role in FXS

Synaptic plasticity refers to the adaptability of synaptic transmission in an activity-dependent manner. Long-term potentiation (LTP) and long-term depression (LTD) are the two most common forms of synaptic plasticity. When the transmission is being strengthened for more than 1 h, it is termed LTP. When the transmission is being weakened for more than 1 h, it is termed LTD. LTP and LTD is the basis for learning and memory, the mGluR-LTD of which plays a central role in FXS pathogenesis (Heller et al., 2014).

Normally, the mGluR-LTD consists of two phases. In the early phase, when mGluR is stimulated, PP2A is instantly activated to dephosphorylate FMRP, derepressing mRNAs targeted by FMRP to cause a rapid synaptic translation burst. In the late phase, the mammalian target of rapamycin (mTOR) is activated. mTOR inhibits PP2A but activates S6K1, which phosphorylates FMRP again to counterbalance the previous translation burst. The overall effect of mGluR stimulation on the surface AMPA receptor is internalization (Nakamoto et al., 2007; Narayanan et al., 2007, 2008; Bassell and Warren, 2008).

In FXS, on the one hand, the signaling cascade from mGluR stimulation to mTOR activation comprises of homer, PIKE, PI3K, PDK1/2, AND Akt, etc. (Santoro et al., 2012). Since FMRP represses the activity of PIKE and PI3K, loss of FMRP results in increased mTOR signaling activity (Sharma et al., 2010). On the other hand, the translation repression at basal state mediated by phosphorylated FMRP is abolished due to FMRP deficiency. As a result, no matter whether there is a stimulus or not, mRNAs targeted by FMRP are always being transcribed actively. The temporarily released translation burst described above is lost, resulting in persistent and increased AMPA receptor internalization. Collectively, the mGluR/mTOR/LTD signaling is exaggerated in FXS (Bassell and Warren, 2008; Santoro et al., 2012).

Theoretically, any miRNA regulating components along the mGluR/mTOR/LTD pathway might play a role in FXS pathogenesis. As described below, accumulating evidence has suggested a bunch of miRNAs could either increase or decrease the activity of mTOR signaling. Although most of them are not directly linked to the pathophysiology of FXS, it is still worth noting that some of them might help unveil novel clues for studying overlapping mechanisms between FXS and other disorders.

Firstly, some miRNAs increase mTOR activity. For example, MiR-125b is an oncogenic miRNA inhibiting the tumor suppressor p53. An important downstream target of p53 is *PIK3CA*, which encodes the p110α subunit of PI3K. As the negative regulation of PIK3CA via p53 is weakened by miR-125b, the mTOR activity is increased (Astanehe et al., 2008; Zeng et al., 2012). miR-451 is also an oncogenic miRNA, which has been implicated in glioma. It may activate the mTOR pathway by inhibiting AMPK signaling in glioma and colorectal cancer cells (Godlewski et al., 2010; Chen et al., 2014). Additionally, both miR-21 and miR-93 are oncogenic and manifest significantly differential expression profiling in ASD. They may increase the mTOR activity by inhibiting the putative target PTEN which is a negative regulator of PI3K (Sarachana et al., 2010; Kawano et al., 2015; Wu Y.E. et al., 2016; Guo X. et al., 2018).

On the contrary, miR-7 and miR-155 are miRNAs downregulating mTOR activity. miR-7 is a tumor suppressor targeting PIK3CD, Akt, and mTOR. PIK3CD is an integral catalytic subunit of PI3K. By inhibiting PI3K, Akt, and mTOR itself, the overall impact of miR-7 on mTOR pathway is suppression (Kefas et al., 2008; Fang et al., 2012). miR-155 is a negative regulator of mTOR via targeting multiple signals including RHEB. It has been implicated both in cancer and autism (Wan et al., 2014; Wu Y.E. et al., 2016).

Moreover, miR-199 seems to play a dual role in mTOR signaling. In cancer cells, it usually acts as a tumor suppressor by downregulating the mTOR pathway activity. In Rett syndrome, miR-199 is a positive regulator of the mTOR pathway activity. However, the MeCP2 deficiency hinders generation of the precursor-miR-199a. As a result, the positive regulation of mTOR signaling via miR-199 is weakened by MeCP2 deficiency, leading to decreased mTOR pathway activity and Rett syndrome phenotypes (Tsujimura et al., 2015).

Last but not least, in turn, the mTOR signaling pathway also regulates the biogenesis and activity of miRNAs. For instance, miR-21 is positively regulated by mTOR, while miR-125b is negatively regulated by mTOR (Ge et al., 2011; Bornachea et al., 2012; Ye et al., 2015).

**Figure 3:**
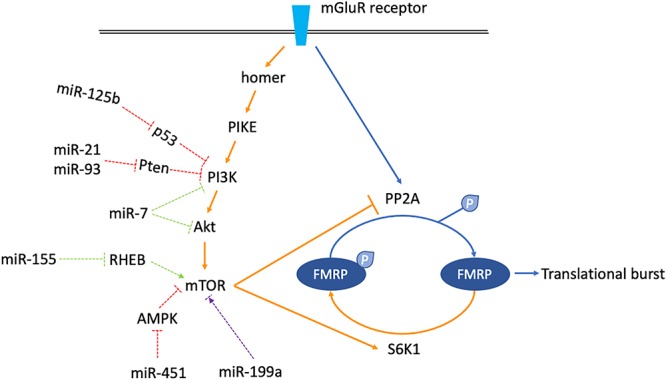
Schematics shown miRNAs which might regulate mGluR/mTOR/LTD signaling. The mGluR/mTOR/LTD consists of two phases. In the early phase, when mGluR receptor is stimulated, the downstream phosphatase PP2A is activated instantly, dephosphorylating FMRP to allow translational burst (arrows are blue colored). In the late phase, activation of the mTOR signaling cascade activates the S6K1 kinase but inhibits the PP2A. FMRP is phosphorylated again, counterbalancing the previous translational burst (arrows are orange colored). miRNAs which might regulate components along the mGluR/mTOR/LTD pathway are classified into three categories. miR-125b, 21, 93, and 451 increase mTOR activity (dashed arrows are red colored). miR-7 and miR-155 decrease mTOR activity (dashed arrows are green colored). miR-199a has a dual function, increasing or decreasing mTOR activity in certain situations (dashed arrows are purple colored).

miRNAs described here are mainly linked to cancer, ASD, and Rett syndrome. Although there is no evidence supporting a direct relationship between them and FXS pathophysiology, it is still possible that several of them may have aberrant functions in FXS. By increasing or decreasing mTOR activity, they might participate in the regulation of mGluR/mTOR/LTD signaling, and further contribute to the shaping of synaptic plasticity (Figure 3). More research on this topic is needed to clarify the linkage between miRNAs and mTOR pathway activity in FXS pathogenesis.

### miRNA May Function as a Broad-Spectrum Therapeutic Agent

The increased mTOR pathway activity is a central pathogenic mechanism shared by FXS, aging, and several other neurological disorders such as autism and tuberous sclerosis, evidenced by reproducible results from yeast to mammalian animal models (Fabrizio et al., 2001; Jia et al., 2004; Johnson et al., 2013; Ebrahimi-Fakhari and Sahin, 2015; Kilincaslan et al., 2017). It is hopeful that the progress of mTOR activity regulation in one disorder may shed light on others.

The mTOR inhibitor has the potential to be a broad-spectrum therapeutic agent. For example, in *FMR1* KO mice, pharmacological inhibition of mTOR pathway rescues multiple FXS phenotypes, including excessive synaptic protein synthesis, persistent AMPA receptor internalization, and increased spine density (Gross et al., 2010). Optimistically, mTOR inhibitors have spawned several clinical trials for FXS (Luo et al., 2016). In aging mice, rapamycin, a classic mTOR inhibitor, was able to slow the aging process in multiple organs (Wilkinson et al., 2012). In tuberous sclerosis patients, everolimus was capable of controlling refractory epilepsy and alleviating autistic symptoms by inhibiting the mTOR pathway activity (Kilincaslan et al., 2017).

Theoretically, miRNAs that inhibit mTOR activity may have therapeutic effects similar to the chemicals described above. In this sense, understanding miRNA mediated mTOR regulation in FXS may help develop new therapeutic agents not only for FXS but also for other disorders where the mTOR dysregulation is an essential pathogenic mechanism.

### miRNA Links FMRP to Alzheimer’s Disease

Alzheimer’s disease (AD) is pathologically featured by the Aβ plaques and neurofibrillary tangles containing hyperphosphorylated tau protein (Zhang et al., 2016). miR-132 and miR-125b are well established FMRP-associated miRNAs regulating synaptic structures and functions (Edbauer et al., 2010). Intriguingly, they are also involved in AD pathogenesis. miR-132 has protective effects on neurons both *in vivo* and *in vitro*, the mechanisms of which include reducing Aβ production and glutamate toxicity, targeting tau modifiers to decrease hyperphosphorylated tau proteins, inhibiting cell apoptosis, and strengthening hippocampal LTP (Smith et al., 2015; Hernandez-Rapp et al., 2016; Salta et al., 2016; Wang et al., 2017; El Fatimy et al., 2018). The expression level of miR-132 in cholinergic nucleus basalis Meynert is stable at early stages but decreases significantly as the disease course develops, suggesting miR-132 may be a facilitator of neurodegeneration (Zhu et al., 2016). In contrast, miR-125b was shown to be a deleterious factor in AD pathogenesis. miRNA profiling of AD brains showed that miR-125b was significantly increased in the hippocampus (Lukiw, 2007). *In vitro* overexpression of miR-125b was shown to induce cell apoptosis, enhance oxidative stress, and promote tau hyperphosphorylation in the cell and animal models of AD (Banzhaf-Strathmann et al., 2014; Jin et al., 2018). As aforementioned, miRNAs assist FMRP mediated translational repression in FXS. Suboptimal ratio and assembly between FMRP, miRNA, and mRNA results in translation dysregulation. Meanwhile, mRNAs of Aβ precursors and Aβ receptors, including NMDARs, mGluR5, and PSD-95, are classic targets negatively regulated by FMRP. Therefore, establishing AD animal models lacking or overexpressing FMRP may help elucidate how FMRP interplays with miR-132 or miR-125b to regulate Aβ related proteins at different disease stages in specific brain regions.

FMRP is also implicated in AD pathogenesis by interacting with lncRNA. It has been controversial whether the BC1 RNA, a lncRNA, functions as an adapter molecule for FMRP in FXS. Some researchers support the view that BC1 directly binds with FMRP to increase its affinity to target mRNAs (Zalfa et al., 2003, 2005; Napoli et al., 2008). Others argue that FMRP directly binds to the target mRNA. The interaction between FMRP and BC1 is non-specific. These two molecules act independently (Iacoangeli et al., 2008; Zhong et al., 2010). Notably, in the context of AD, this controversial issue seems to be reconciled to some extent. In the Tg2576 AD mice model, when FMRP binds to *APP* mRNA, *APP* translation is suppressed, subsequently producing less Aβ protein. However, when BC1 binds to FMRP, the interaction between FMRP and *APP* mRNA is disrupted, resulting in active *APP* translation and increased Aβ production, which further causes the spatial learning and memory deficits (Zhang et al., 2018). This study is a good example where FMRP indeed binds directly with either BC1 RNA or target mRNA. However, this binding does not necessarily include BC1 RNA and target mRNA simultaneously. Therefore, investigating how BC1 and other lncRNAs, *APP*, and FMRP interact with each other at synapsis may help understand the pathogenesis of both AD and FXS.

### FMRP Regulates Stem Cell Fate via miRNA

The first evidence indicating FMRP may regulate germline stem cell fate via the miRNA pathway came in 2007. In drosophila, The interaction between FMRP and Ago1 may facilitate maintenance but suppress differentiation of the germline stem cells (Yang et al., 2007). FMRP may also function via the bantam miRNA to regulate the germline stem cell fate (Yang et al., 2009). In testicle cells, FMRP was found to be associated with a bunch of miRNAs including miR-383, which is mainly expressed in spermatogonia. miR-383 is negatively regulated by FMRP during spermatogenesis, failure of which may contribute to male infertility (Tian et al., 2013). In the brain, miR-510, located near the fragile site on X chromosome, was associated with CGG expansion into full mutation in the neurons derived from mesenchymal stem cells. Bioinformatics analysis indicated that enhanced miR-510 expression might facilitate CGG expansion via regulating target genes such as VHL and PPP2R5E (Fazeli et al., 2018). In mice embryonic NPC, the upregulation of miR-130b associates with decreased FMRP level and increased NPC proliferation tendency (Gong et al., 2013). The expression level of miR-181a, which translationally represses the expression of GluA2, a subunit of AMPA receptors, was increased in human NPCs derived from induced pluripotent stem cells. Since cells lacking the GluA2 subunit were more prone to differentiation, miR-181a may affect cell fate by interacting with the AMPA signaling pathway (Achuta et al., 2018). According to these data, it is fair to say that the FXS stem cell model provides an excellent monogenic platform for studying how miRNAs are involved in stem cell fate regulation, especially in testis and brain.

## Closing Remarks and Outlook

In summary, Non-coding RNA participates in FXS pathophysiology via multiple aspects. The RNAi mechanism provides a RNA-based model for explaining the epigenetic silencing of *FMR1*. The miRNA mainly facilitates FMRP’s role as a translational repressor, controlling axon growth, shaping synaptic plasticity, and regulating endogenous FMRP level. The lncRNA also plays non-negligible roles (Table 1). Besides, understanding how non-coding RNA functions in FXS may help uncover converging mechanisms shared by FXS and related disorders. This list may well include FXTAS, ASD, Rett syndrome, AD, tuberous sclerosis, infertility, cancer, and aging, etc. In the future, studies dissecting the non-coding RNA profiling temporally and spatially are needed to identify the non-coding RNA regulation pattern in specific neurodevelopmental or neurodegenerative stages. The non-coding RNA that may function as a nexus between FXS and related disorders should be given more attention. As such research findings will help unveil converging mechanisms shared by different diseases and further contribute to the development of a broad-spectrum non-coding RNA therapeutic agent.

**Table 1:** Non-coding RNA involved in FXS pathogenesis.

Type	Mechanism	Reference
miR-125b	Facilitate FMRP to regulate NMDA receptor subunit expression	Edbauer et al., 2010
	Increase mTOR activity	Astanehe et al., 2008; Zeng et al., 2012
miR-181d	Regulate axon elongation	Wang et al., 2015
miR-130b	Regulate FMRP expression	Gong et al., 2013; Suhl et al., 2015
FMR4	lncRNA with anti-apoptotic function	Khalil et al., 2008
	Modulate NPC proliferation	Peschansky et al., 2016
FMR5,6	lncRNA may be used as biomarkers	Pastori et al., 2014
TUG1	lncRNA prevents axonal growth	Guo Y. et al., 2018
miR-124a	Regulate astroglial glutamate transportation	Morel et al., 2013
BC1	lncRNA may serve as an adaptor molecule of FMRP	Zhang et al., 2018

## Author Contributions

All authors contributed to the manuscript writing, revision, and figure design.

## Conflict of Interest Statement

The authors declare that the research was conducted in the absence of any commercial or financial relationships that could be construed as a potential conflict of interest.
